# ColdstartMHDTI: integrating biomolecular pretraining and attention-based heterogeneous graph learning for drug–target interaction prediction

**DOI:** 10.3389/fchem.2026.1846850

**Published:** 2026-07-06

**Authors:** Hongyang Yang, Xiucai Ye, Hui Han, Tetsuya Sakurai

**Affiliations:** Department of Computer Science, University of Tsukuba, Tsukuba, Japan

**Keywords:** cross attention, drug-target interaction, heterogeneous-network, large language model, multimodalities

## Abstract

**Motivation:**

Accurate drug–target interaction (DTI) prediction remains difficult for underexplored drugs and targets, especially when available interaction evidence is sparse. Existing approaches often focus either on pairwise molecular representations or on heterogeneous biomedical graph topology, making it difficult to effectively integrate structure-derived representations with multi-relational contextual evidence.

**Result:**

We propose ColdstartMHDTI, a two-stage framework for heterogeneous-graph-based DTI prediction that integrates sequence-derived structural representations with local and global relational information. Specifically, drug SMILES and target sequences are encoded by pretrained transformer models, while one-hop heterogeneous relations are captured through self-supervised DistMult embeddings. These representations are then fused through a meta-path-guided module that models ordered meta-path instances and aggregates them with cross-attention for interaction scoring. Across two benchmark datasets, ColdstartMHDTI shows consistent improvements under warm-start and entity-disjoint settings, with particularly strong performance for underexplored drugs and targets. It also remains robust under more imbalanced evaluation protocols with 1:5 and 1:10 positive-to-negative ratios. In addition to standard classification performance, ColdstartMHDTI supports candidate prioritization for downstream screening and evidence-guided hypothesis generation. Case studies on ESR1, EGFR, and Parkinson’s disease further demonstrate its practical utility, with the Parkinson’s disease analysis additionally highlighting strong per-drug target ranking performance.

## 1 Introduction

Drugs are typically developed to act on specific molecular targets; however, they often interact with multiple unintended targets, which may lead to off-target effects and unexpected phenotypes (Huang et al., 2024). Conversely, such polypharmacology also provides opportunities for drug repurposing. For example, mebendazole, an anthelmintic drug used to treat parasitic infections (Keiser and Utzinger, 2008), has shown antitumor activity in lung cancer by suppressing cancer cell proliferation (Mukhopadhyay and Sasaki, 2002). These observations highlight the importance of accurately identifying DTIs to support both drug discovery and repositioning (Doudican et al., 2008; Hopkins, 2008).

Traditional computational methods for DTI prediction mainly include ligand-based modeling and molecular docking (Shang et al., 2022; Yamanishi et al., 2010). Ligand-based approaches rely on sufficient known active compounds and often degrade when labeled interactions are scarce, while docking methods require high-quality three-dimensional target structures that are frequently unavailable or fail to capture relevant binding conformations. To alleviate these limitations, recent studies have increasingly adopted data-driven approaches based on graph neural networks and large language models.

GNN-based methods model drugs, targets, and related biological entities as nodes in graphs and learn embeddings by aggregating neighborhood information (Fu et al., 2020; Himmelstein et al., 2017). Early studies integrated drug chemical similarity and target genomic similarity information to infer drug–target interaction networks (Yamanishi et al., 2008). Although effective, such similarity-based methods rely on shallow features and fixed network structures, limiting their ability to capture higher-order semantics and handle cold-start scenarios. To overcome these issues, GraphDTA (Nguyen et al., 2021) introduced end-to-end representation learning by encoding drugs as molecular graphs and targets as amino-acid sequences, achieving substantial performance improvements. Subsequently, heterogeneous GNN models such as MHGNN (Li et al., 2023) incorporated meta-path–based semantic attention to integrate multi-type biological entities, including diseases and side effects, and explicitly modeled higher-order relationships. However, these approaches mainly depend on network topology and do not fully exploit rich molecular or sequence features, which constrains their generalization to unseen drugs or targets.

In parallel, LLM-based approaches have been proposed to learn expressive representations directly from molecular structures and target sequences (Grisoni, 2023; Ramos et al., 2024; Zhang et al., 2024; Zhao et al., 2024). MolTrans (Huang et al., 2021) applied the Transformer architecture to model semantic interactions between drug substructures and target subsequences. More recently, large-scale pretrained target language models, such as ESM-2, have demonstrated strong representation capacity for downstream DTI prediction, particularly under cold-start settings (Ahmed and Ansari, 2024; Singh et al., 2023). Building on this line of work, ColdstartCPI (Zhao et al., 2025) combined LLM-derived representations with inductive bias from induced-fit theory, substantially improving generalization to novel drugs or targets. Despite these advances, most LLM-based methods focus primarily on drug and target sequences and incorporate limited information from other biologically relevant entities, such as diseases or side effects. Effectively integrating LLM representations with heterogeneous biological networks therefore remains an open challenge.

To address these limitations, we propose ColdstartMHDTI, a two-stage DTI prediction framework that combines self-supervised pretraining with meta-path–guided heterogeneous graph learning. Specifically, we extract pretrained representations for drugs and targets from SMILES strings and amino-acid sequences using ChemBERTa and ESM-2, respectively, to capture sequence-derived structural information. For diseases and side effects, we learn DistMult embeddings from their one-hop relations with drugs and targets, providing local relational information from the heterogeneous network (Ye et al., 2021). We then use the pretrained embeddings of all entity types as initial node features and perform meta-path–guided message integration with an encoder that operates on ordered meta-path instances: each instance is modeled as a short token sequence with positional embeddings, and information is fused through instance-level and meta-path-level cross-attention, while residual connections preserve pretrained signals. Finally, an MLP predictor estimates interaction probabilities under both warm-start and entity-disjoint cold-start settings. Experiments on two benchmark datasets show that ColdstartMHDTI consistently outperforms strong baselines, with particularly large gains in cold-start scenarios. We further validate top-ranked predictions with literature evidence and structure-based analyses, supporting the practical utility of our framework for drug repurposing. In summary, our key contributions are as follows:

1. We propose ColdstartMHDTI, a two-stage framework that integrates sequence-derived representations with local relational information from heterogeneous biomedical networks and captures global multi-hop dependencies through a meta-path–guided encoder. Meta-path instances are modeled as ordered token sequences with positional embeddings and fused using instance-level and meta-path-level cross-attention, with residual connections retaining pretrained signals.
2. We focus on cold-start DTI prediction and conduct entity-disjoint evaluation under drug-cold and target-cold settings, followed by additional 1:5 and 1:10 imbalanced evaluations to assess robustness under sparse candidate distributions. To quantify the contribution of disease/side-effect relationships, we further perform a controlled comparison against pairwise DTI baselines that rely solely on molecular and sequence representations.
3. We demonstrate the practical utility of ColdstartMHDTI through case studies with evidence-based verification. In particular, the Parkinson’s disease case study highlights strong per-drug target ranking performance, supporting candidate prioritization for downstream virtual screening and drug repurposing.

## 2 Dataset construction

In this study, two benchmark datasets comprising different kinds of heterogeneous data, namely, Luo’s dataset (Luo et al., 2017) and Mei’s dataset (Li et al., 2023), were used to benchmark our method against other state-of-the-art methods for DTI prediction. For clarity, Luo’s dataset and Mei’s dataset are hereafter referred to as Hetero-A and Hetero-B, respectively.

Luo’s dataset is composed of four types of nodes (drugs, targets, diseases, and side-effects) and six types of edges (drug–target, drug–drug, target–target, drug–disease, target–disease, and drug–side-effect). Because the Luo dataset contains duplicate targets, we cleaned it by removing duplicate nodes and edges. After deduplication, it contains 1,996 nodes and 1,895,443 edges (more detailed information is provided in Table 1).

**TABLE 1:** Data construction information table.

Types	Items	Hetero-A numbers	Hetero-A resources	Hetero-B numbers	Hetero-B resources
Node	Drug (D)	708	DrugBank (Version 3.0)	2,214	DrugBank (Version 5.1.8)
	Target (T)	1,493	HPRD (Release 9)	1968	UniProtKB (Release 2022)
	Disease (I)	5,603	CTD (2013)	7,205	CTD (2021)
	Side-effect (S)	4,192	SIDER (Version 2)	3,935	SIDER (Version 4)
Edge	D-T	1920	DrugBank (Version 3.0)	8,750	DrugBank (Version 5.1.8)
	D-D	10,036	DrugBank (Version 3.0)	1,091,870	DrugBank (Version 5.1.8)
	D-I	199,214	CTD (2013)	542,970	CTD (2021)
	D-S T-T T-I	80,164 7,363 1,596,745	SIDER (Version 2) HPRD (Release 9) CTD (2013)	1,042,629 456,592 2,922,064	SIDER (Version 4) STRING (Version 11) CTD (2021)

Mei’s dataset is a biological knowledge graph integrating biomedical information from 14 databases, developed for relational learning. It consists of three components: links, attributes, and entity metadata. Links (e.g., target–target and drug–target interactions) capture relationships between biological entities. Attributes provide annotation fields for entities and relations, while entity metadata include names, types, and synonyms. Overall, the dataset contains 15,322 unique nodes and 5,126,875 edges (see Table 1 for details).

**FIGURE 1:**
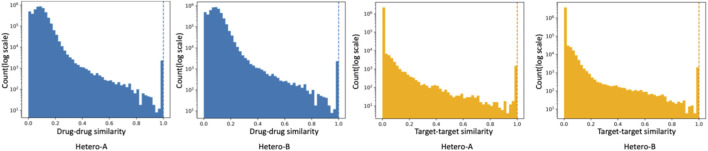
Drug-drug and target-target similarity-score distributions on datasets Hetero-A and Hetero-B.

In DTI prediction, high pairwise similarity among drugs or among targets risks overfitting and can yield optimistic evaluation. We therefore assessed pairwise similarity in each dataset. Specifically, we computed Tanimoto coefficients from drug SMILES strings (Rogers and Hahn, 2010) and used them as structural similarity for each drug-drug pair (DDP). For targets, we computed normalized DIAMOND blastp bit scores (Buchfink et al., 2021) from sequences and used them as similarity for each target-target pair (TTP). Figure 1 shows the similarity distributions of DDPs and TTPs on the Hetero-A and Hetero-B datasets. Most drug-drug similarities are near zero: 99.8873% (Hetero-A) and 99.9166% (Hetero-B) of DDPs are below 0.5. For targets, 99.9753% (Hetero-A) and 99.9677% (Hetero-B) of TTPs are below 0.5, with the bulk of the mass concentrated near zero.

## 3 Model design

In ColdstartMHDTI, the framework comprises two main modules: a pretraining module and a fusion module. The pretraining module learns the initial representations of drugs, targets, diseases, and side effects. Building on these initial features, the fusion module integrates multi-entity information and captures global network information from the heterogeneous graph, yielding the final fused representations for drugs and targets.

### 3.1 Pretraining modules

**FIGURE 2:**
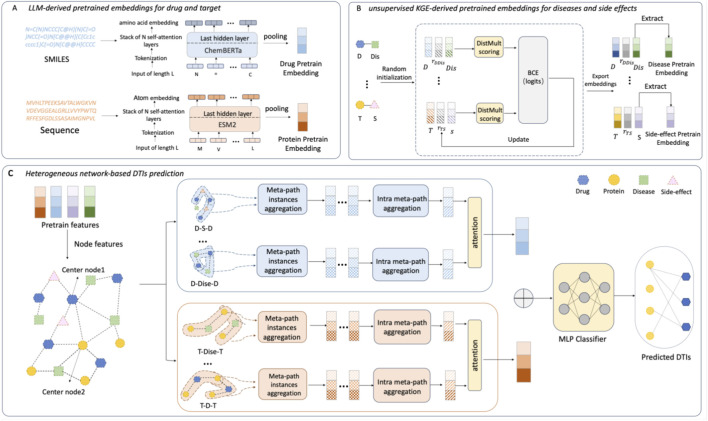
Overview of the ColdstartMHDTI workflow **(A)** LLM-pretrained embeddings for drugs and targets extracted from SMILES and amino-acid sequences. **(B)** Unsupervised DistMult embeddings for diseases and side effects learned from the heterogeneous network. **(C)** Meta-path integration and DTI prediction modules that fuse entity representations and meta-path information to predict DTIs.

To initialize node features across entity types, we use LLM encoders for drugs and targets and an unsupervised DistMult pipeline for diseases and side effects (Figures 2A,B). Specifically, ChemBERTa and ESM-2 encode drug SMILES and target sequences into pretrained representations that capture sequence-derived features. In parallel, DistMult learns pretrained embeddings for diseases and side effects from their relations with drugs and targets in the heterogeneous network, providing local relational information for downstream graph modeling.

For drugs and targets with molecular and sequence information, we initialize their representations using pretrained ChemBERTa (Ahmad et al., 2022) on SMILES and pretrained ESM-2 (Lin et al., 2023) on target sequences (Figure 2A). Given a SMILES string with (*n*_*d*_) tokens, ChemBERTa yields token embeddings ( $M_{d}\in R^{n_{d}\times 384}$ ). For a target sequence of length (*n*_*p*_), ESM-2 yields token embeddings ( $M_{p}\in R^{n_{p}\times 1024}$ ). We then apply mean pooling over tokens and amino acids to obtain fixed-length vectors ( $m_{d}\in R^{1\times 384}$ ) and ( $m_{p}\in R^{1\times 1024}$ ).

For diseases and side effects that lack explicit molecular or sequence features, we obtain their representations using an unsupervised DistMult-based knowledge graph embedding model trained with a link-prediction objective (Figure 2B) (Yang et al., 2015; Ye et al., 2021). Specifically, based on known associations, we represent the data as a set of typed triples {(*h,r,t*)}, where h and t denote the head and tail entities with embedding vectors (*e*_*h*_) and (*e*_*t*_), and (*r*) denotes the relation type with an embedding vector (*w*_*r*_). The entity set includes drugs, targets, diseases, and side effects, while relation types correspond to observed pairwise associations (e.g., target–disease, target–side effect). For a triple (*h,r,t*), DistMult computes the score using Equation 1:

$$S=\sum_{i=1}^{k}{\left[e_{h}\right]}_{i}{\left[w_{r}\right]}_{i}{\left[e_{t}\right]}_{i}$$ (1)

where k is the embedding dimension and ${\left[\cdot \right]}_{i}$ denotes the *i*th element. For each triple, we assign label y ∈ {0, 1}, where y = 1 for a positive triple and y = 0 for a negative triple. Using the DistMult scores as the logit, we minimize the BCE-with-logits loss using Equation 2:

$$L_{KGE}=-\sum_{j=1}^{\left|D\right|}\left[y_{j}\log\;\sigma \left(s_{j}\right)+\left(1-y_{j}\right)\log\;\left(1-\sigma \left(s_{j}\right)\right)\;\right]$$ (2)

where D denotes the set of positive and negative triples, and j denotes the index of a triple in D . We update all entity and relation embeddings by backpropagation with stochastic gradient descent. After training, we export the learned embedding vectors e for disease and side-effect as their pretrained embeddings. Because DistMult captures only direct (one-hop) relations between nodes, the resulting disease and side-effect pretrained embeddings encode local network information from the heterogeneous graph.

Finally, all four types of embeddings (drugs, targets, diseases, and side effects) are mapped into a unified 128-dimensional space by an MLP and used as initial node features in the constructed heterogeneous biological network.

### 3.2 Meta-path integration module

Before learning meta-path embeddings, we first construct a heterogeneous biological network comprising four node types (drugs, targets, diseases, and side effects) and six relation types among them. We then define a set of meta-paths as type-level sequences that capture multi-hop semantic relationships in the heterogeneous network. For example, the drug–disease–drug meta-path reveals how drugs are connected through shared diseases. Following Wang et al. (2025), we removed meta-paths that are overly dense or semantically diffuse, and prioritized those that provide rich connectivity and strong semantic cues. The final set of meta-paths is: drug–drug, drug–target–drug, drug–target–target–drug, drug–target–disease–target–drug, target–target, target–drug–target, target–drug–drug–target, and target–drug–disease–drug–target.

The meta-path embedding module consists of three major components: meta-path-instance aggregation, intra-meta-path aggregation, and inter-meta-path aggregation. Figure 2C illustrates the overall workflow of the Meta-path Embedding module.

#### 3.2.1 Meta-path instance aggregation

**FIGURE 3:**
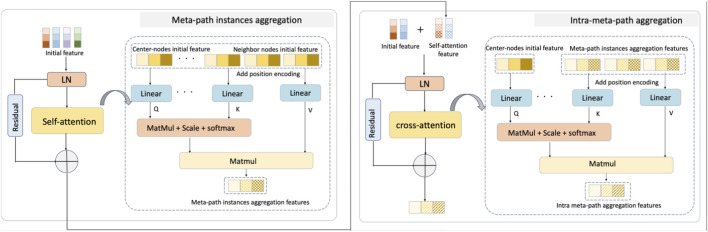
Meta-path instances aggregation module and intra-meta-path aggregation module.

For a center node i∈V and a meta-path type p , we collect an ordered set of meta-path instances that start from i . We treat each instance as a short token sequence (Figure 3, left), and use cross-attention to integrate information from the center node and its neighbors within the instance.

For a center node i , meta-path type p induces $R_{p,i}$ ordered instances. The *r*-th instance is defined in Equation 3 as

$$\Gamma_{p,i}=\left(i,v_{1},\ldots ,v_{L_{p}-1}\right)$$ (3)

where $L_{p}$ is the length of meta-path type p ; for example, $L_{p}=3$ for the drug-target-drug meta-path; $v_{j}$ denotes the j -th node along the r -th instance of meta-path type starting from center node i .

Let $x_{v}\in R^{d}$ denote the feature of node v . We stack node features in the instance order to form the matrix in Equation 4:

$$X_{p,i}=\left[x_{i};\;x_{v_{1}};\ldots ;x_{v_{L_{p}-1}}\right]\in R^{L_{p}\times d}$$ (4)

We then add a learnable positional embedding as shown in Equation 5. $P_{p}\in R^{L_{p}\times d}$ shared by all instances of type p:

$$Z_{p,i}=X_{p,i}+P_{p}$$ (5)

Following Figure 3, we apply pre-norm multi-head cross-attention with a residual connection as shown in Equation 6:

$$H_{p,i}=Z_{p,i}+\text{MHA}\left(\mathrm{LN}\left(Z_{p,i}\right)\right)$$ (6)

where LN (·) denotes layer normalization applied to each token vector and MHA (·) denotes multi-head cross-attention. The residual connection retains the original token features, while cross-attention aggregates information along the meta-path instance.

Finally, we take the center-node output as the instance message, as defined in Equation 7:

$$m_{p,i}=H_{p,i}\left[0\right]\in R^{d}$$ (7)

where [0] denotes the row corresponding to the center node i .

#### 3.2.2 Intra-meta-path aggregation

For a given meta-path type p , the center node i corresponds to a set of instance messages ${\left\{m_{p,i}\right\}}_{r=1}^{\left(R_{p,i}\right)}$ . We then aggregate these messages to obtain a meta-path–specific representation for i . We stack them into the matrix defined in Equation 8:

$$M_{p,i}=\left[m_{p,i}^{1};\;\ldots ;m_{p,i}^{R_{p,i}}\right]\in R^{R_{p,i}\times d}$$ (8)

As shown in Figure 3 (intra-meta-path aggregation), we use the center node’s initial feature *x*_*i*_ as the query to aggregate the instance messages, as shown in Equation 9:

$$h_{p,i}=x_{i}+\text{MHA }\left(Q=\mathrm{LN}\left(x_{i}\right),K=M_{p,i},V=M_{p,i}\right)\in R^{d}$$ (9)

Here, $Q\in R^{1\times d}$ and $K,V\in R^{R_{p,i}\times d}$ . When batching nodes with different $R_{p,i}$ , we apply an attention mask to ignore padded instances. The residual connection retains the center node’s original representation and augments it with the meta-path–level aggregated signal.

#### 3.2.3 Inter-meta-path aggregation

For a center node i , let $P_{i}$ denote the set of meta-path types defined for the type of node i . After intra-meta-path aggregation, we obtain a meta-path–specific embedding $h_{p,i}\in R^{d}$ for each meta-path type $p\in P_{i}$ . We then apply an attention mechanism to compute the importance of each meta-path for node *i*, as shown in Equation 10:

$$h_{i}=\sum_{p\in P_{i}}\beta_{p,i}h_{p,i}\in R^{d}$$ (10)

where $\beta_{p,i}$ is the softmax-normalized attention weight of meta-path type p for node i ; $\beta_{p,i}\geq 0$ and the weights over all meta-path types for node i sum to 1.

### 3.3 Training

After meta-path aggregation, we obtain a drug embedding $h_{u}\in R^{d}$ and a target embedding $h_{v}\in R^{d}$ from the Meta-path Embedding Module. We model their interaction using an MLP on the element-wise product, as shown in Equation 11:

$$x=h_{u}⊙{\;h}_{v}$$ (11)

The MLP outputs a scalar logit according to Equation 12:

$$s=w_{2}^{T}\text{ReLU}\left(W_{1}x+b_{1}\right)+b_{2}\in R$$ (12)

where $W_{1}\in R^{h\times d}$ , $w_{2}\in R^{h}$ , and ${\;b}_{1}\in R^{h}$ , ${\;b}_{2}\in R$ are learnable parameters. Given a mini-batch with $y^{\left(n\right)}\in \left\{0,1\right\}$ , we optimize the binary cross-entropy loss with logits using Equation 13:

$$L_{BCE}=-\frac{1}{B}\sum_{n=1}^{B}\left[-y^{\left(n\right)}\log\;\sigma \left(s^{\left(n\right)}\right)-\left(1-y^{\left(n\right)}\right)\log\;(1-\sigma \left(s^{\left(n\right)}\right)\right]$$ (13)

where σ (·) is the sigmoid function. All learnable parameters in the Meta-path Embedding Module and the MLP predictor are optimized end-to-end *via* back-propagation.

### 3.4 Experimental setup and evaluation metrics

#### 3.4.1 Dataset splitting and implementation details

We evaluated ColdstartMHDTI under one warm-start setting and two cold-start settings (drug-cold and target-cold) using five pre-generated splits with different random seeds. In the drug-cold setting, drugs in the training set do not appear in the validation or test sets, whereas in the target-cold setting, targets in the training set do not appear in the validation or test sets.

Under the standard 1:1 protocol, each split was constructed with a balanced positive-to-negative ratio of 1:1. In the warm-start setting, the data in each split were further divided into training, validation, and test sets at a ratio of 7:2:1. In the two cold-start settings, the same 1:1 positive-to-negative ratio was retained while enforcing entity disjointness across splits. Negative samples were drawn from drug–target pairs that were not recorded as known interactions in the latest database release after excluding all currently known positive pairs. Because experimentally validated non-interacting pairs are rarely available in current public benchmarks, these sampled pairs were treated as putative negatives. The validation set was used for early stopping and model selection, and all reported results were obtained on the test set.

To further assess robustness under sparser candidate distributions, we additionally constructed imbalanced evaluation settings with positive-to-negative ratios of 1:5 and 1:10. Specifically, for each warm-start, drug-cold-start, and target-cold-start split, all positive samples were preserved and additional negative pairs were sampled so that each split followed a 1:5 or 1:10 positive-to-negative ratio, respectively.

We trained the model for up to 200 epochs using AdamW with a learning rate of 1e-4, batch size 256, and dropout 0.3. The hidden dimension is 128 with eight attention heads, and we sample 200 neighbors per node. Early stopping is applied with a patience of 10 based on the validation set, and the checkpoint with the best validation performance is used for test evaluation. Experiments were run on an NVIDIA Tesla V100-PCIE-32GB GPU (CUDA 12.1, PyTorch 2.3.1).

#### 3.4.2 Evaluation metrics

We evaluated each model using standard metrics for binary DTI prediction, including accuracy (ACC), F1 score (F1), Matthews correlation coefficient (MCC), and the areas under the receiver operating characteristic and precision–recall curves (AUC and AUPR). Unless otherwise specified, these metrics were computed on the held-out test set by pooling all drug–target pairs within each split for micro-level evaluation. The final results are reported as the mean ± standard deviation across five runs.

## 4 Results and discussion

### 4.1 Performance under warm-start and cold-start settings

#### 4.1.1 Standard balanced evaluation

To demonstrate the advantages of ColdstartMHDTI, we compare it with eight state-of-the-art (SOTA) baselines on two large-scale public datasets under three evaluation settings (one warm-start and two cold-start settings), using a standard 1:1 balanced positive-to-negative sampling protocol.

Among the baselines, IMCHGAN (Li et al., 2022), KGE_NFM (Ye et al., 2021), CCL-ASPS (Tian et al., 2024), DTINet (Luo et al., 2017), MAGNN (Li et al., 2023), and DHGT-DTI (Wang et al., 2025) are heterogeneous biomedical network-based DTI models that explicitly capture drug/target–disease and drug/target–side-effect associations. To assess the contribution of disease/side-effect information, we additionally evaluated DrugBAN (Bai et al., 2023) and ColdstartCPI (Zhao et al., 2025), which model DTIs as drug–target pair prediction using molecular and sequence representations without incorporating disease/side-effect relationships. For fair comparison, all these baselines adopt the same training and test sets as ColdstartMHDTI.

IMCHGAN (Li et al., 2022) leverages GAT to learn embeddings of drugs and targets from DTI heterogeneous networks, then fuses the embeddings through an attention mechanism.

KGE_NFM (Ye et al., 2021) combines multi-relational knowledge-graph embeddings with collaborative filtering to jointly model drugs and targets, improving DTI prediction accuracy, interpretability, and cold-start generalization.

DHGT-DTI (Wang et al., 2025) predicts DTIs on a dual-view heterogeneous network by fusing local neighbor semantics and global meta-path dependencies to obtain drug/target representations for interaction scoring.

MAGNN (Li et al., 2023) learns heterogeneous node representations by meta-path–guided message aggregation with hierarchical attention over meta-path instances and types.

CCL-ASPS (Tian et al., 2024) learns joint drug–target representations by collaboratively contrasting cross-modal embeddings and adaptively sampling informative pairs *via* a self-paced strategy.

DrugBAN (Bai et al., 2023) models drug–target interactions by applying bidirectional attention between drug atoms and target residues to learn a joint pair representation for prediction.

ColdstartCPI (Zhao et al., 2025) fuses pretrained Mol2Vec and ProtTrans features with a Transformer to model drug–target interactions for cold-start DTI prediction.

On the Hetero-A dataset (Table 2), ColdstartMHDTI achieves the best results under all three evaluation settings. Under the warm-start setting, ColdstartMHDTI attains an average AUC of 0.974 ± 0.009 and AUPR of 0.979 ± 0.010, outperforming the second-best method, DHGT-DTI (AUC 0.960 ± 0.007; AUPR 0.967 ± 0.008). When moving to the drug-cold setting, most baselines degrade substantially relative to warm-start, whereas ColdstartMHDTI remains robust with AUC 0.954 ± 0.011 and AUPR 0.956 ± 0.012; compared with the second-best DHGT-DTI (AUC 0.909 ± 0.016; AUPR 0.904 ± 0.011), this corresponds to improvements of 0.043 in AUC and 0.052 in AUPR, together with a higher MCC (0.793 vs. 0.672). In the target-cold setting, ColdstartMHDTI again ranks first across all metrics, achieving AUC 0.944 ± 0.011 and AUPR 0.948 ± 0.012; compared with DHGT-DTI (AUC 0.923 ± 0.012; AUPR 0.945 ± 0.017), the gains increase to 0.021 in AUC, alongside higher AUPR, ACC, F1, and MCC, indicating strong generalization to unseen targets.

**TABLE 2:** Comparison with the latest methods on Hetero-A.

Scenarios	Methods	ACC	AUC	AUPR	F1	MCC
Warm-start	DrugBAN	0.862 ± 0.010	0.916 ± 0.013	0.910 ± 0.012	0.866 ± 0.029	0.725 ± 0.020
	ColdstartCPI	0.875 ± 0.024	0.928 ± 0.022	0.936 ± 0.016	0.876 ± 0.020	0.750 ± 0.023
	IMCHGAN	0.790 ± 0.012	0.886 ± 0.021	0.902 ± 0.014	0.798 ± 0.023	0.639 ± 0.033
	KGE_NFM	0.855 ± 0.015	0.925 ± 0.014	0.929 ± 0.015	0.848 ± 0.019	0.711 ± 0.028
	CCL_ASPS	0.875 ± 0.013	0.936 ± 0.011	0.943 ± 0.016	0.873 ± 0.024	0.728 ± 0.020
	MAGNN	0.805 ± 0.014	0.875 ± 0.012	0.858 ± 0.012	0.816 ± 0.017	0.613 ± 0.019
	DHGT-DTI	0.902 ± 0.022	0.960 ± 0.007	0.967 ± 0.008	0.899 ± 0.012	0.804 ± 0.015
	*ours*	**0.929 ± 0.014**	**0.974 ± 0.009**	**0.979 ± 0.010**	**0.923 ± 0.015**	**0.831 ± 0.017**
Drug-cold start	DrugBAN	0.724 ± 0.011	0.740 ± 0.019	0.778 ± 0.027	0.615 ± 0.016	0.406 ± 0.024
	ColdstartCPI	0.801 ± 0.010	0.869 ± 0.017	0.865 ± 0.030	0.776 ± 0.012	0.607 ± 0.005
	IMCHGAN	0.636 ± 0.019	0.705 ± 0.031	0.384 ± 0.028	0.358 ± 0.024	0.198 ± 0.022
	KGE_NFM	0.693 ± 0.007	0.774 ± 0.025	0.442 ± 0.022	0.416 ± 0.011	0.386 ± 0.025
	CCL_ASPS	0.712 ± 0.011	0.761 ± 0.028	0.805 ± 0.011	0.780 ± 0.013	0.312 ± 0.012
	MAGNN	0.619 ± 0.014	0.833 ± 0.018	0.819 ± 0.019	0.551 ± 0.016	0.335 ± 0.011
	DHGT-DTI	0.836 ± 0.012	0.909 ± 0.016	0.904 ± 0.011	0.824 ± 0.021	0.672 ± 0.019
	*ours*	**0.889 ± 0.010**	**0.954 ± 0.011**	**0.956 ± 0.012**	**0.889 ± 0.012**	**0.793 ± 0.021**
Target-cold start	DrugBAN	0.683 ± 0.015	0.791 ± 0.018	0.712 ± 0.025	0.466 ± 0.025	0.312 ± 0.024
	ColdstartCPI	0.786 ± 0.017	0.852 ± 0.015	0.856 ± 0.012	0.781 ± 0.028	0.601 ± 0.015
	IMCHGAN	0.521 ± 0.029	0.674 ± 0.021	0.336 ± 0.051	0.273 ± 0.072	0.101 ± 0.037
	KGE_NFM	0.524 ± 0.026	0.715 ± 0.012	0.424 ± 0.015	0.315 ± 0.024	0.231 ± 0.028
	CCL_ASPS	0.691 ± 0.024	0.783 ± 0.039	0.732 ± 0.037	0.644 ± 0.024	0.471 ± 0.023
	MAGNN	0.715 ± 0.014	0.842 ± 0.021	0.795 ± 0.029	0.618 ± 0.021	0.414 ± 0.022
	DHGT-DTI	0.836 ± 0.021	0.923 ± 0.012	0.945 ± 0.017	0.861 ± 0.014	0.665 ± 0.015
	*ours*	**0.869 ± 0.027**	**0.944 ± 0.011**	**0.948 ± 0.012**	**0.877 ± 0.021**	**0.748 ± 0.019**

The bold font indicates the classifiers that work best.

On Hetero-B (Table 3), ColdstartMHDTI achieves the best performance under warm-start evaluation. For drug cold-start and target cold-start settings, DHGT-DTI results are not included because the authors have not released an implementation and cold-start numbers on Hetero-B are not reported in the original study. We therefore compare against the remaining baselines that can be reproduced under the same data splits and evaluation protocol, and report results transparently with missing entries indicated as “/”. Compared with a recent reproducible baseline, ColdstartCPI, ColdstartMHDTI achieves average improvements of 0.087 in AUROC and 0.085 in AUPR under cold-start evaluation, demonstrating consistent gains over reproducible baselines on Hetero-B.

**TABLE 3:** Comparison with the latest methods on Hetero-B.

Scenarios	Methods	ACC	AUC	AUPR	F1	MCC
Warm-start	DrugBAN	0.882 ± 0.010	0.946 ± 0.013	0.950 ± 0.012	0.891 ± 0.029	0.759 ± 0.020
	ColdstartCPI	0.885 ± 0.024	0.958 ± 0.022	0.966 ± 0.016	0.897 ± 0.020	0.800 ± 0.023
	IMCHGAN	0.850 ± 0.012	0.870 ± 0.021	0.886 ± 0.014	0.793 ± 0.023	0.618 ± 0.033
	KGE_NFM	0.852 ± 0.015	0.915 ± 0.014	0.915 ± 0.015	0.838 ± 0.019	0.686 ± 0.028
	CCL_ASPS	0.887 ± 0.013	0.946 ± 0.011	0.953 ± 0.016	0.883 ± 0.024	0.747 ± 0.020
	MAGNN	0.908 ± 0.014	0.919 ± 0.012	0.900 ± 0.012	0.675 ± 0.017	0.540 ± 0.019
	DHGT-DTI	0.920 ± 0.026	0.970 ± 0.017	0.974 ± 0.005	0.913 ± 0.012	0.821 ± 0.015
	*ours*	**0.927 ± 0.014**	**0.975 ± 0.012**	**0.976 ± 0.010**	**0.924 ± 0.015**	**0.847 ± 0.017**
Drug-cold start	DrugBAN	0.701 ± 0.011	0.731 ± 0.019	0.737 ± 0.027	0.600 ± 0.016	0.356 ± 0.024
	ColdstartCPI	0.781 ± 0.010	0.837 ± 0.017	0.843 ± 0.030	0.732 ± 0.012	0.571 ± 0.005
	IMCHGAN	0.617 ± 0.019	0.672 ± 0.031	0.681 ± 0.028	0.303 ± 0.024	0.172 ± 0.022
	KGE_NFM	0.661 ± 0.007	0.752 ± 0.025	0.760 ± 0.022	0.400 ± 0.011	0.361 ± 0.025
	CCL_ASPS	0.703 ± 0.011	0.751 ± 0.028	0.785 ± 0.011	0.641 ± 0.012	0.301 ± 0.022
	MAGNN	0.611 ± 0.014	0.801 ± 0.018	0.814 ± 0.019	0.429 ± 0.016	0.329 ± 0.011
	DHGT-DTI	**—**	**—**	**—**	**—**	**—**
	*ours*	**0.839 ± 0.010**	**0.920 ± 0.011**	**0.925 ± 0.012**	**0.835 ± 0.012**	**0.680 ± 0.021**
Target-cold start	DrugBAN	0.683 ± 0.015	0.791 ± 0.018	0.712 ± 0.025	0.466 ± 0.025	0.312 ± 0.024
	ColdstartCPI	0.761 ± 0.017	0.812 ± 0.015	0.816 ± 0.012	0.710 ± 0.028	0.557 ± 0.015
	IMCHGAN	0.520 ± 0.029	0.581 ± 0.021	0.304 ± 0.051	0.221 ± 0.022	0.100 ± 0.027
	KGE_NFM	0.530 ± 0.026	0.693 ± 0.012	0.384 ± 0.015	0.300 ± 0.010	0.211 ± 0.018
	CCL_ASPS	0.691 ± 0.024	0.783 ± 0.039	0.732 ± 0.037	0.644 ± 0.024	0.471 ± 0.023
	MAGNN	0.620 ± 0.014	0.772 ± 0.021	0.781 ± 0.029	0.411 ± 0.021	0.324 ± 0.022
	DHGT-DTI	**—**	**—**	**—**	**—**	**—**
	*ours*	**0.816 ± 0.027**	**0.904 ± 0.011**	**0.906 ± 0.012**	**0.811 ± 0.021**	**0.642 ± 0.019**

The bold font indicates the classifiers that work best.

Moreover, on the Hetero-A dataset, we compare ColdstartMHDTI and DHGT-DTI with DrugBAN and ColdstartCPI. Across the warm-start, drug-cold-start, and target-cold-start settings, ColdstartMHDTI and DHGT-DTI, which explicitly model drug/target–disease and drug/target–side-effect associations, achieve mean AUROC/AUPR of 0.930/0.957 and 0.938/0.961, respectively. In contrast, DrugBAN and ColdstartCPI, which use only drug and target representations without modeling disease/side-effect associations, obtain lower mean AUROC/AUPR of 0.815/0.883 and 0.800/0.886. These results suggest that disease/side-effect associations provide complementary information that improve DTI prediction, particularly under cold-start evaluation.

#### 4.1.2 Imbalanced evaluation under 1:5 and 1:10 negative ratios

While the standard 1:1 balanced protocol provides a controlled benchmark for model comparison, it does not reflect the strong sparsity of practical DTI screening, where true interactions are rare among candidate drug–target pairs. To further evaluate robustness under more realistic class imbalance, we conducted additional experiments under positive-to-negative ratios of 1:5 and 1:10. The corresponding results are summarized in Table 4.

**TABLE 4:** Performance of ColdstartMHDTI under 1:1, 1:5, and 1:10 positive-to-negative evaluation settings on Hetero-A and Hetero-B.

Scenarios	Methods	Ratio	ACC	AUC	AUPR	F1	MCC
Warm-start	Hetero-A	1:1	0.929 ± 0.014	0.974 ± 0.009	0.979 ± 0.010	0.923 ± 0.015	0.831 ± 0.017
		1:5	0.951 ± 0.017	0.967 ± 0.014	0.908 ± 0.011	0.853 ± 0.016	0.802 ± 0.012
		1:10	0.964 ± 0.015	0.949 ± 0.018	0.878 ± 0.020	0.826 ± 0.015	0.768 ± 0.019
	Hetero-B	1:1	0.927 ± 0.014	0.975 ± 0.012	0.976 ± 0.010	0.924 ± 0.015	0.847 ± 0.017
		1:5	0.956 ± 0.021	0.971 ± 0.022	0.914 ± 0.025	0.866 ± 0.019	0.825 ± 0.018
		1:10	0.964 ± 0.015	0.936 ± 0.011	0.882 ± 0.016	0.843 ± 0.024	0.747 ± 0.020
Drug-cold start	Hetero-A	1:1	0.889 ± 0.010	0.954 ± 0.011	0.956 ± 0.012	0.889 ± 0.012	0.793 ± 0.021
		1:5	0.930 ± 0.014	0.944 ± 0.021	0.845 ± 0.022	0.790 ± 0.018	0.735 ± 0.019
		1:10	0.948 ± 0.017	0.939 ± 0.016	0.808 ± 0.027	0.748 ± 0.012	0.690 ± 0.021
	Hetero-B	1:1	0.839 ± 0.010	0.920 ± 0.011	0.925 ± 0.012	0.835 ± 0.012	0.680 ± 0.021
		1:5	0.925 ± 0.011	0.916 ± 0.013	0.860 ± 0.017	0.784 ± 0.014	0.695 ± 0.022
		1:10	0.944 ± 0.013	0.903 ± 0.021	0.830 ± 0.018	0.752 ± 0.019	0.652 ± 0.021
Target-cold start	Hetero-A	1:1	0.869 ± 0.027	0.944 ± 0.011	0.948 ± 0.012	0.877 ± 0.021	0.748 ± 0.019
		1:5	0.917 ± 0.017	0.934 ± 0.015	0.832 ± 0.012	0.770 ± 0.028	0.703 ± 0.025
		1:10	0.938 ± 0.023	0.919 ± 0.022	0.795 ± 0.015	0.730 ± 0.023	0.655 ± 0.014
	Hetero-B	1:1	0.816 ± 0.027	0.904 ± 0.011	0.906 ± 0.012	0.811 ± 0.021	0.642 ± 0.019
		1:5	0.908 ± 0.026	0.899 ± 0.012	0.842 ± 0.015	0.754 ± 0.010	0.655 ± 0.018
		1:10	0.931 ± 0.024	0.874 ± 0.039	0.812 ± 0.037	0.726 ± 0.024	0.610 ± 0.023

As shown in Table 4, increasing the negative ratio from 1:1 to 1:5 and 1:10 consistently made the task more challenging on both Hetero-A and Hetero-B under warm-start, drug-cold-start, and target-cold-start settings. Although ACC generally increased with larger negative ratios, this increase should be interpreted cautiously, as the model could achieve higher accuracy simply by predicting more samples as negatives under increasingly imbalanced evaluation sets. In contrast, AUPR, F1, and MCC showed clearer declines, especially in the two cold-start settings, indicating that identifying true DTIs became more difficult under sparser candidate distributions. Nevertheless, ColdstartMHDTI maintained relatively strong AUPR and MCC across both datasets and all settings, suggesting that its predictive advantage is not restricted to the standard 1:1 balanced protocol.

### 4.2 Ablation experiment validation

To further validate the contribution of each component, we conducted ablation experiments. In these experiments, we trained the ColdstartMHDTI model without specific components using the same hyperparameters, training settings, and five-fold cross-validation splits. Specifically, WODSPretrain replaces the pretrained disease and side-effect features with one-hot features while keeping all other modules unchanged; WODTPretrain replaces the pretrained drug and target features with one-hot features under the same setting; and WOHG removes the meta-path embedding module and instead uses a multi-head GCN to learn drug and target representations.

**FIGURE 4:**
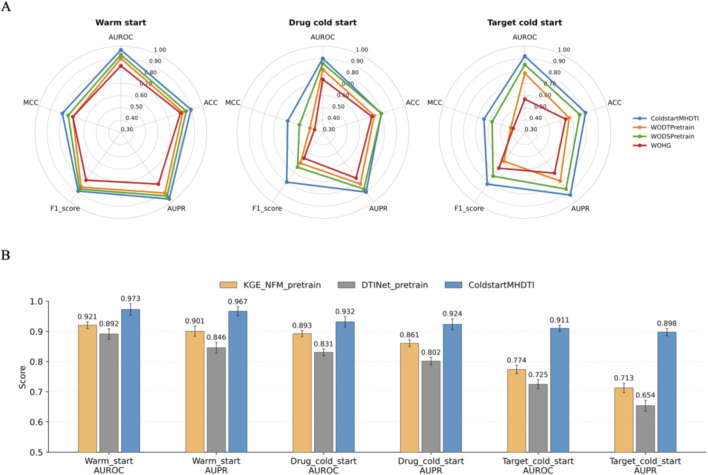
Ablation Experiment Validation Result Diagram **(A)** Ablation study under warm-start, drug-cold-start, and target-cold-start settings. **(B)** Performance comparison among ColdstartMHDTI, KGE_NFM_pretrain, and DTINet_pretrain.

Figure 4A summarizes the results on Hetero-A under warm-start, drug-cold-start, and target-cold-start using radar charts of AUROC, AUPR, ACC, F1, and MCC. Overall, ColdstartMHDTI achieves the best performance across all three settings, indicating that each component contributes to the final predictive accuracy. Importantly, the benefit of the two pretraining components becomes more evident under cold-start evaluation. When removing DTPretrain (WODTPretrain), performance drops markedly in both drug-cold-start and target-cold-start, suggesting that pretrained SMILES- and sequence-derived representations provide transferable information that is critical for generalization to unseen drugs or targets. Removing DSPretrain (WODSPretrain) also consistently degrades cold-start performance, indicating that disease and side-effect pretraining supplies complementary relational information that helps connect unseen entities to the observed graph through shared disease/side-effect associations. Among the ablations, removing the meta-path integration module (WOHG) results in the largest degradation, further highlighting that meta-path–guided aggregation is necessary to effectively propagate and integrate these pretrained signals in the heterogeneous network.

Furthermore, to further examine the impact of ColdstartMHDTI’s meta-path integration module, we evaluate two representative DTI baselines trained on the same dataset: KGE_NFM (Ye et al., 2021) and DTINet (Luo et al., 2017). We replace the original feature extractors in KGE_NFM and DTINet with the same pretrained drug and target features used in ColdstartMHDTI, while keeping all other model components and the training procedure unchanged. We refer to these variants as KGE_NFM_pretrain and DTINet_pretrain, respectively.

Figure 4B reports the results on Hetero-A dataset under the warm-start, drug-cold-start, and target-cold-start settings. Using the same drug and target representations as input, ColdstartMHDTI consistently achieves the best overall performance across metrics, indicating that its meta-path–guided embedding and predictor better exploit heterogeneous relationships than KGE_NFM and DTINet.

### 4.3 Comparison of end-to-end variants

Additionally, to compare offline pretrained embeddings with task-adaptive, end-to-end representations, we replace the offline pretraining stage in ColdstartMHDTI with end-to-end trainable encoders. We thus obtain three variants, DSTrans-Tuning, ProtTrans-Tuning, and DrugTrans-Tuning.

In DSTrans-Tuning, we keep the drug and target initial features unchanged and retain all other settings, but replace the pretrained disease and side-effect embeddings learned by the unsupervised DistMult stage. Specifically, we attach the DistMult component to the DTI predictor in an end-to-end manner, so that disease and side-effect embeddings are updated jointly with the DTI training objective rather than being fixed pretrained features.

In ProtTrans-Tuning and DrugTrans-Tuning, we similarly keep all other settings unchanged and replace only the initial representations of targets or drugs, respectively. Instead of using fixed, offline-extracted pretrained embeddings, we integrate ProtTrans (for target amino-acid sequences) or DrugTrans (for drug SMILES strings) as end-to-end encoder modules in the downstream DTI prediction framework. Both encoders are initialized with weights pretrained on large-scale corpora and are fine-tuned jointly with the DTI prediction objective using Adam, yielding task-adaptive target or drug representations.

We evaluate these variants on the Hetero-A dataset under the same experimental settings, and summarize the results in Table 5. DSTrans-Tuning performs comparably to ColdstartMHDTI under warm-start but degrades under drug-cold-start and target-cold-start settings, which may be due to its larger number of trainable parameters that increases overfitting and weakens generalization. ProtTrans-Tuning shows a similar pattern: it performs well under warm-start but drops under both cold-start settings, which can be explained by two factors. First, fine-tuning may shift pretrained parameters away from broadly useful representations. Second, the larger parameter budget increases the risk of overfitting, thereby reducing generalization. DrugTrans-Tuning is close to ProtTrans-Tuning under warm-start but decreases further in both cold-start settings, suggesting that end-to-end training of large pretrained encoders tends to overfit and generalize less well to unseen drugs or targets.

**TABLE 5:** Comparison of end-to-end tuning variants on Hetero-A.

Scenarios	Dataset	AUROC	AUPR
Warm-start	DSTrans-tuning	0.951	0.958
	ProtTrans-tuning	0.937	0.939
	DrugTrans-tuning	0.928	0.921
	ColdstartMHDTI	0.974	0.979
Drug-cold start	DSTrans-tuning	0.831	0.847
	ProtTrans-tuning	0.815	0.834
	DrugTrans-tuning	0.788	0.810
	ColdstartMHDTI	0.954	0.956
Target-cold start	DSTrans-tuning	0.857	0.864
	ProtTrans-tuning	0.824	0.808
	DrugTrans-tuning	0.795	0.771
	ColdstartMHDTI	0.944	0.948

### 4.4 Impact of meta-path aggregation methods on performance

Given a meta-path instance, the aggregation layer encodes the target node by combining meta-path based neighbors with their sequential and semantic information. Different aggregation strategies can affect meta-path representations and downstream performance. We evaluate six methods for learning meta-path-instance embeddings: average, linear, max-pooling, neighbor-linear, GraphTransformer (Dwivedi and Bresson, 2021), and the proposed cross attention mechanism. This comparison allows us to quantify the advantage of the proposed cross attention mechanism over these alternative aggregation schemes:

1. Average: Mean-pools the node features along the meta-path to obtain a single embedding for each instance.
2. Linear: Averages the node features along the meta-path, then applies a linear projection to obtain a multi-head embedding for each instance.
3. Max-pooling: Applies a linear transform to each node feature and then takes a dimension-wise maximum across the meta-path instance, retaining the strongest signal per feature dimension.
4. Neighbor-linear: Uses only the feature of the center node on the meta-path and projects it through a linear layer to obtain the final instance embedding.
5. GraphTransformer: Treats each meta-path instance as a directed chain, merges all chains into a single graph, and applies multi-head graph attention for message passing. The terminal-node representation is used as the instance embedding.
6. Cross attention: our method.

**FIGURE 5:**
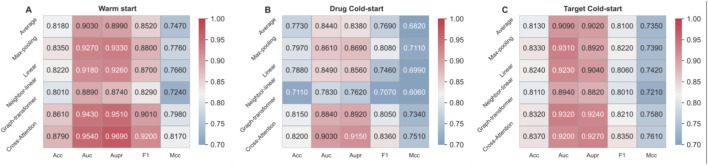
Performance of different meta-path aggregation strategies under three scenarios. **(A)** Warm-start setting. **(B)** Drug-cold-start setting. **(C)** Target-cold-start setting. The heatmaps compare different aggregation strategies in terms of ACC, AUC, AUPR, F1, and MCC.

Figure 5 summarizes the performance of the six methods in terms of Acc, AUC, AUPR, F1, and MCC. Under the warm-start and drug cold-start settings, the cross attention achieves the best overall performance; it attains the highest ACC, AUROC, and AUPR, while maintaining competitive F1 and MCC. In the target cold-start setting, max-pooling and GraphTransformer perform slightly better on Acc and AUC, whereas cross attention remains best on AUPR; F1 and MCC are comparatively close among the top methods. These trends suggest that cross attention is more effective at ranking positives under class imbalance, which may stem from its explicit positional embeddings that encode meta-path order relative to the target node and its attention restricted to the sequence dimension, enabling stable computation and helping the model learn fine-grained semantics on short meta-paths. Conversely, in target cold-start, stronger distribution shift in target representations may favor more conservative aggregation (e.g., pooling or GraphTransformer), improving Acc/AUC while leaving AUPR less affected.

### 4.5 Case study and prediction analysis

To assess the reliability of ColdstartMHDTI, we conducted case studies on Parkinson’s disease and two cancer-related targets, ESR1 and EGFR.

#### 4.5.1 Validation on Parkinson’s disease

Parkinson’s disease is a prevalent neurological degenerative disorder that predominantly affects middle-aged and elderly individuals and manifests motor symptoms such as tremors, muscle rigidity, bradykinesia, and postural instability (Albin and Grotewold, 2023). Simultaneously, it is accompanied by non-motor symptoms like anxiety, sleep disturbances, and cognitive deterioration, which significantly impact the quality of life. Although there is no cure currently available, medication can be efficacious in alleviating symptoms and enhancing daily functionality. This section will delve into several drugs for Parkinson’s disease and their target associations.

In the Hetero-A dataset, we selected four Parkinson’s drugs: Cabergoline, Ropinirole, Apomorphine, and Bromocriptine. We initially removed all DTI records associated with these drugs. Subsequently, positive samples were formed with the remaining associated data, and a corresponding number of negative samples was randomly chosen to build a training set for conducting the experiment. Once the model training was completed, we tested the relationship between each drug and other targets, organizing them by predicted scores, starting with the highest values. The results of the top 10 predicted scores are presented in Table 6.

**TABLE 6:** Top 10 candidate targets related to four drugs.

Drug	Rank	Target	Evidence	Rank	Target	Evidence
Cabergoline (DB00248)	1	P21728 DDR1	DrugBank	6	P28335 HTR2C	DrugBank
	2	P28223 HTR2A	DrugBank	7	P35372 OPRM1	Unconfirmed
	3	P08912 CHRM5	Unconfirmed	8	P08172 CHRM2	Unconfirmed
	4	P14416 DRD2	DrugBank	9	P41595 HTR2B	DrugBank
	5	P08908 HTR1A	DrugBank	10	P35462 DRD3	DrugBank
Ropinirole (DB00268)	1	P35348 ADRA1A	DrugBank	6	P35368 ADRA1B	DrugBank
	2	P35462 DRD3	DrugBank	7	P21554 CNR1	Unconfirmed
	3	P28223 HTR2A	DrugBank	8	Q13639 HTR4	DrugBank
	4	P25021 HRH2	Unconfirmed	9	P14416 DRD2	DrugBank
	5	P28335 HTR2C	DrugBank	10	Q9H3N8 HRH4	Unconfirmed
Apomorphine (DB00714)	1	P25100 ADRA1D	Unconfirmed	6	P18825 ADRA2C	DrugBank
	2	P08913 ADRA2A	DrugBank	7	P35462 DRD3	DrugBank
	3	P11229 CHRM1	Unconfirmed	8	P21917 DRD4	DrugBank
	4	P35368 ADRA1B	Unconirmed	9	P28223 HTR2A	DrugBank
	5	P18089 ADRA2B	DrugBank	10	P14416 DRD2	DrugBank
Bromocriptine (DB01200)	1	P35462 DRD3	DrugBank	6	P08913 ADRA2A	DrugBank
	2	P3536 ADRA1B	DrugBank	7	P28222 HTR1B	DrugBank
	3	P28221 HTR1D	DrugBank	8	P25100 ADRA1D	DrugBank
	4	P18089 ADRA2B	DrugBank	9	P35348 ADRA1A	DrugBank
	5	P21728 DRD1	DrugBank	10	P28223 HTR2A	DrugBank

**FIGURE 6:**
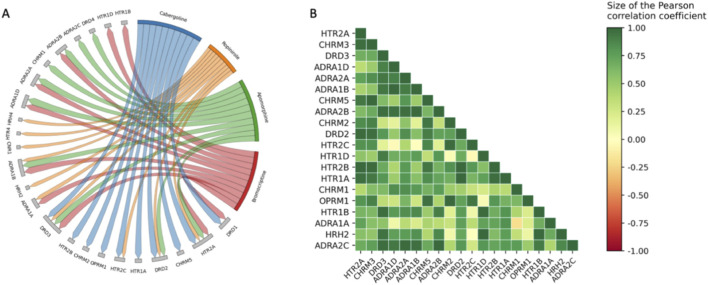
Case study results **(A)** Chordal diagram of four drugs and their predicted targets. **(B)** Correlation heatmaps for predicting targets. The colors of the scale represent the size of the Pearson correlation coefficient. The greener the color, the stronger the positive correlation (closer to +1.00), while the redder the color, the stronger the negative correlation (closer to −1.00).

As can be observed from Table 6, for the drug Cabergoline, Ropinirole and Apomorphine, seven of the top 10 associations could be verified from the DrugBank database. For Bromocriptine, all top 10 associations are validated. We plotted the associations in Table 6 as a chord diagram (Figure 6A), where the line weights in the chord diagram represent the predicted scores of the model. As can be seen from the figure, there is a considerable overlap in the targets of the action of these four drugs for treating Parkinson’s disease. This indicates that there is some resemblance in the target selection of these four drugs, which might imply that they operate through similar biological pathways or mechanisms. Specifically, the overlap of targets among drugs may reflect their effects on the same or related neurotransmitter systems during the treatment of Parkinson’s disease. Additionally, this overlap may also offer clues for researchers to consider these targets when developing new drugs to enhance treatment efficacy.

In the domain of DTI prediction, the well-known “guilt-by association” hypothesis is frequently adhered to, signifying that similar drugs are associated with similar targets. Based on this, we hypothesized that there is a correlation between the candidate targets predicted by the four Parkinson’s drugs. To do this, we computed the Pearson correlation coefficient heatmap for the predicted 20 targets (Figure 6B). It can be noted from the thermal maps that there is a close correlation between these targets.

#### 4.5.2 Validation on ESR1 and EGFR

To further confirm ColdstartMHDTI’s reliability and address challenges in anticancer drug discovery, we selected ESR1 (Brett et al., 2021; Rej et al., 2023) and EGFR (Corvaja et al., 2024; Sha and Lee, 2024)—two cancer-associated targets—as case studies and predicted candidate drugs. Specifically, on Dataset B, we eliminated all DTI records associated with these targets. Candidates were ranked by the prediction score, and we conducted a comprehensive literature search to assess the accuracy of the top 100 predicted interactions. We also estimated binding affinity with AutoDock Vina (Eberhardt et al., 2021) for the top-100 and bottom-100 pairs. To validate our predicted drug–target interactions that are not included in existing records, we further performed blind docking using CB-Dock2 (Liu et al., 2022) with default settings, and reported the top-ranked binding pose and score returned by the server.

**FIGURE 7:**
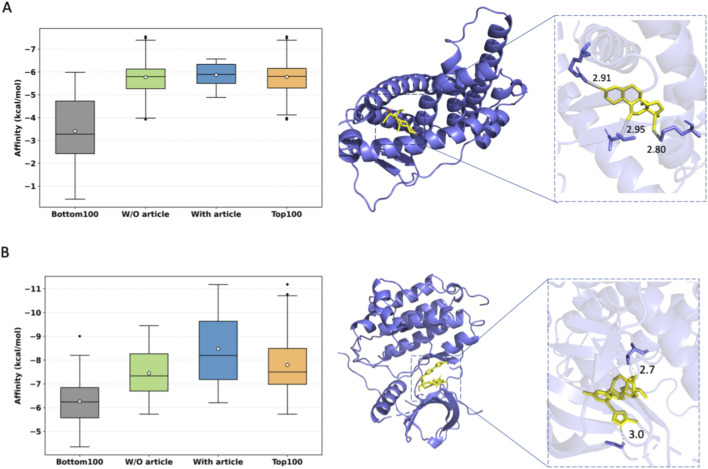
Case Study on ESR1 and EGFR **(A)** Docking affinity distributions for bottom 100, top 100, and top-100 subsets (with article vs. w/o article) against ESR1; predicted pose of DB00294–ESR1 is shown. **(B)** Corresponding results for EGFR; predicted pose of DB15822–EGFR is shown.

Figure 7 shows boxplots of docking binding affinities for the top 100 and bottom 100 drugs ranked by our predicted interaction scores with ESR1 and EGFR. For ESR1, the mean pocket docking scores computed by AutoDock Vina (more negative indicates stronger predicted binding) were −7.797 kcal/mol for the top 100 candidates, including −8.478 kcal/mol for the 56 literature-supported compounds (“with article”) and −7.447 kcal/mol for the remaining 44 unvalidated compounds (“w/o article”). In contrast, the bottom 100 candidates averaged −6.252 kcal/mol. A Kruskal–Wallis test (Kruskal and Wallis, 1952) confirmed significantly stronger docking for the top-ranked set than for the bottom-ranked set (). Among the w/o article candidates, DB00294 achieved the strongest docking score (−9.011 kcal/mol). Additional blind docking with CB-Dock2 yielded a score of −9.4 kcal/mol and indicated that 66 ligand atoms of DB00294 lie within 4.0Å of ESR1 residues; the zoomed-in view in Figure 7A highlights representative non-covalent contacts, including van der Waals neighbors within 3.0 Å and potential hydrogen-bond/polar interactions. $p=2.78\times 10^{-17}$ .

For EGFR, the mean AutoDock Vina pocket scores were −5.785 kcal/mol for the top 100 candidates, with −5.870 kcal/mol for the 12 literature-supported compounds and −5.775 kcal/mol for the remaining 88 unvalidated compounds, whereas the bottom 100 averaged −2.651 kcal/mol. The separation between the top- and bottom-ranked sets was significant by a Kruskal–Wallis test ( $p=2.78\times 10^{-17}$ ). Among the unvalidated candidates, DB15822 achieved the strongest docking score (−7.148 kcal/mol). CB-Dock2 docking for DB15822 produced a score of −8.6 kcal/mol and reported that 88 ligand atoms lie within 4.0Å of EGFR residues; the zoomed-in view shows representative short-range contacts within 3.0 Å.

## 5 Discussion and conclusion

In this work, we propose ColdstartMHDTI, a drug–target interaction prediction framework specifically designed to address cold-start scenarios. By integrating LLM-derived representations of drugs and targets with meta-path-based modeling on heterogeneous biomedical networks, ColdstartMHDTI captures both intrinsic biomolecular features and high-order relational information.

Across two benchmark datasets and three evaluation settings, ColdstartMHDTI achieved consistently strong performance, with particularly notable gains in cold-start scenarios. These results suggest that combining pretrained biomolecular representations with heterogeneous relational information improves DTI prediction when available interaction evidence is sparse, thereby enhancing generalization to previously unseen drugs or targets.

From a methodological perspective, this study demonstrates the value of jointly modeling biomolecular representations and heterogeneous relational evidence for cold-start DTI prediction. By combining sequence-level representations with network-level relational signals, ColdstartMHDTI can capture both intrinsic molecular properties and higher-order biomedical relationships without relying solely on handcrafted features, pairwise interaction histories, or single-source structural information.

In practical terms, ColdstartMHDTI can provide valuable computational support for early-stage drug discovery, particularly in scenarios where experimental validation is costly and limited. By prioritizing potential drug–target interactions, the proposed framework enables efficient candidate selection in large chemical and target spaces, which is essential for rational drug design and virtual screening. Furthermore, the predicted interactions can be integrated into downstream validation pipelines, including molecular docking, biochemical assays, and preclinical studies, as commonly adopted in previous works on small-molecule drug discovery (He et al., 2019; Liu et al., 2023; Qin et al., 2024; Wang et al., 2019; Xu et al., 2021; Ye et al., 2022; Zhou et al., 2023; Zhu et al., 2025). In this context, ColdstartMHDTI may serve as an upstream screening tool that facilitates hypothesis generation and reduces the search space for experimental validation. Overall, this discussion highlights the potential of ColdstartMHDTI to bridge data-driven prediction and experimental drug discovery workflows, thereby supporting more efficient identification of promising drug candidates.

Despite these promising results, several limitations should be acknowledged. First, ColdstartMHDTI mainly focuses on binary drug–target interaction prediction and does not explicitly distinguish pharmacological action types, such as inhibition, activation, agonism, or antagonism, which may limit its mechanistic specificity. Second, although case studies and docking analyses provide supportive evidence, the model does not explicitly incorporate curated binding-site or three-dimensional structural information, and its top-ranked predictions still require further experimental validation. Addressing these limitations will help improve the mechanistic interpretability, structural reliability, and translational applicability of ColdstartMHDTI.

Future work will focus on developing explanation strategies to better clarify how different features, meta-paths, and relational structures contribute to the final predictions. In addition, incorporating pharmacological action labels, such as inhibition, activation, agonism, and antagonism, may provide more detailed mechanistic guidance beyond binary DTI prediction. Another promising direction is to combine ColdstartMHDTI with curated binding-site information, structure-based modeling, and generative approaches, which could improve structural interpretability and extend the framework toward *de novo* drug design and molecular optimization.

In summary, ColdstartMHDTI provides an effective framework for drug–target interaction prediction under cold-start conditions. By integrating pretrained biomolecular representations with heterogeneous relational modeling, it offers a promising direction for data-driven drug discovery.

## Data Availability

The original data files and core codes supporting the conclusions of this article are available at https://github.com/koino1/ColdstartMHDTI. Further inquiries can be directed to the corresponding author.
